# Simple Experimental Methods for Determining the Apparent Focal Shift in a Microscope System

**DOI:** 10.1371/journal.pone.0134616

**Published:** 2015-08-13

**Authors:** Benjamin P. Bratton, Joshua W. Shaevitz

**Affiliations:** 1 Department of Physics, Princeton University, Princeton, NJ, United States of America; 2 Lewis-Sigler Institute for Integrative Genomics, Princeton University, Princeton, NJ, United States of America; Pennsylvania State Hershey College of Medicine, UNITED STATES

## Abstract

Three-dimensional optical microscopy is often complicated by a refractive index mismatch between the sample and objective lens. This mismatch causes focal shift, a difference between sample motion and focal-plane motion, that hinders the accuracy of 3D reconstructions. We present two methods for measuring focal shift using fluorescent beads of different sizes and ring-stained fluorescent beads. These simple methods are applicable to most situations, including total internal reflection objectives and samples very close to the interface. For distances 0–1.5 *μ*m into an aqueous environment, our 1.49-NA objective has a relative focal shift of 0.57 ± 0.02, significantly smaller than the simple *n*_2_/*n*_1_ approximation of 0.88. We also expand on a previous sub-critical angle theory by means of a simple polynomial extrapolation. We test the validity of this extrapolation by measuring the apparent focal shift in samples where the refractive index is between 1.33 and 1.45 and with objectives with numerical apertures between 1.25 and 1.49.

## Introduction

**Fig 1:**
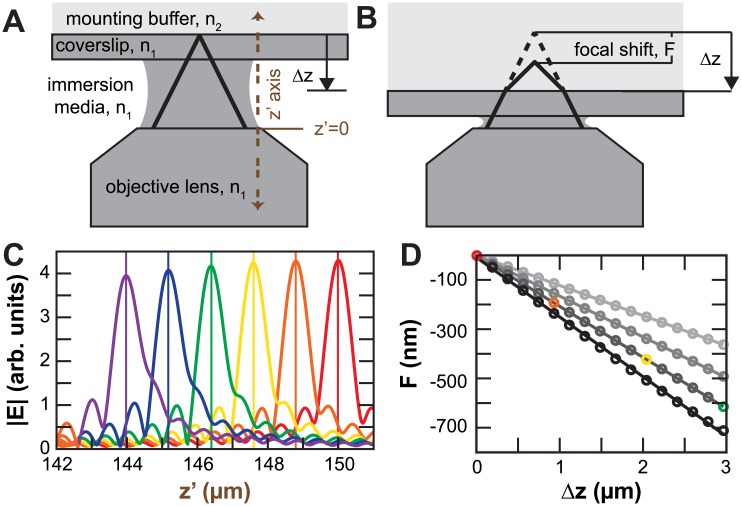
Schematic of idealized imaging system and focal shift. (A,B) When the sample and the objective have difference refractive indices, moving the sample closer to the objective does not move the effective focal plane the same distance. Brown dashed line indicates the z’ axis and z’ = 0 at the surface of the objective. In this schematic, *F* and Δ*Z* have opposite signs, corresponding to a situation of *n*_2_ < *n*_1_. (C) Plots of intensity of electric field along the optical axis z’ for different positions of the *n*_1_ − *n*_2_ interface (Eq 1, NA = 1.2). Colors indicate sample positions from 150 *μ*m (red) through 147 *μ*m (green) to 145 *μ*m (purple). (D) Focal shifts are proportional to sample displacements. Colored circles refer to peak positions from panel C. Darker grays indicate larger NAs (0.13, 1.0, 1.2, 1.3).

In high-resolution biological fluorescence microscopy, one often images samples embedded in a medium with a different refractive index than the microscope objective lens. This difference in refractive index results leads to a phenomenon known as focal shift where movement of the sample relative to the objective lens by an amount Δ*z* shifts the actual focal plane in the sample by a different amount Δ*z* + *F* (Fig 1A and 1B). The mounting medium for common biological applications is aqueous and has a lower refractive index (*n*_2_ ≈ 1.33) than the objective lens material (*n*_1_ ≈ 1.51). In these situations, *F* has the opposite sign of Δ*z* and the focal plane moves less than the anticipated amount. When the mounting media has a higher refractive index than the objective, for example when using a water-immersion objective lens with a sample mounted in plastic, the focal shift *F* has the same sign as Δ*z* and the effective focal plane is moved more than the anticipated amount.

A variety of theoretical and experimental studies have demonstrated that focal shift is proportional to the displacement, *F* ∝ Δ*z* [1, 2]. *F* can be calculated by examining the electric field generated by a plane-wave propagating through a lens and the index-mismatch interface in the *z*′-direction [2]

$$\begin{array}{l} E\left(z^{\prime }\right)=\frac{i\;k_{1}\;f}{2}\int_{0}^{\Omega }\sqrt{\text{cos}\theta_{1}}\;\text{sin}\theta_{1}\\ \;\;\;\;\;\;\;\times \text{exp}\left[i\;\left(f\;-\;d\;\right)\left(k_{1}\;\text{cos}\theta_{1}\;-\;k_{2}\;\text{cos}\theta_{2}\right)\right]\\ \;\;\;\;\;\;\;\times \left(\tau_{s}\;+\;\tau_{p}\;\text{cos}\theta_{2}\right)\text{exp}\left(-i\;k_{2}\;z^{\prime }\;\text{cos}\theta_{2}\right)\;d\theta_{1} \end{array}$$ (1)

where *f* is the lens focal length, *d* is the distance from the lens to the interface, *k*_*j*_ is the wave number in the first (*j* = 1) and second (*j* = 2) medium, *θ*_1_, *θ*_2_ are the polar angles in the two media, Ω is the semi-aperture angle of the lens, and *τ*_*s*_ and *τ*_*p*_ are the Fresnel transmission coefficients for the two polarizations of light. Fig 1D shows the linearity of *F* with respect to Δ*z* for sample displacements between 0 and 1.5 *μ*m relative to the index-mismatch plane (*λ* = 500 nm, *n*_1_ = 1.515, *n*_2_ = 1.33). This integral sums over the contributions from all the rays that make up the numerical aperture. Larger numerical apertures yield larger deviations between the displacement of the focal plane and the displacement of the sample, corresponding to a smaller relative focal shift (Fig 1D). We define the relative focal shift as

$$\begin{array}{c} \alpha \equiv (\Delta z+F)/\Delta z. \end{array}$$ (2)

In practice, to convert sample motion into true focal plane displacement, one only requires the conversion factor *α* and the sample displacement Δ*z*.

The observed 3D intensity profile from a fluorescence measurement depends on the 3D illumination profile, the size and shape of the sample, and the microscope point-spread function–a function that describes the observed intensity profile from a point source. The focal shift can have a variety of practical implications depending on the imaging modality used. For epifluorescence illumination, the illumination profile is designed to be essentially uniform for the entire sample volume so that aberrations to this from focal shift can be ignored. For applications in which a focused beam of light is scanned through a sample for the purposes of fluorescence excitation, e.g. in confocal fluorescence microscopy and stimulated emission-depletion microscopy, the increasingly-large effect of focal shift with increasing numerical aperture (NA) is mitigated by total internal reflection at the plane of index-mismatch (assuming *n*_2_ < *n*_1_ as is typical for biological applications). For TIR objectives (NA > *n*_2_), super-critical rays are totally internally reflected and do not contribute to the focused illumination spot or the excitation focal shift. Fluorescence emission, in contrast, affects both epifluorescence and beam-scanning modalities and requires a larger focal-shift correction than for excitation because the emitted light does not undergo total internal reflection at the interface. One consequence of this is a changing collection efficiency with depth for beam-scanning applications. A recent model including both illumination and collection focal shift was published for the case of 3D structured illumination microscopy [3].

A few labs have attempted to measure focal shift on specific imaging systems. These methods often agree well with numerical simulations and differences are assumed to be due to system-specific aberrations. However, these methods are incapable of measuring the focal shift very close to the coverslip, the regime of interest for studying thin sections of tissue or bacterial cells. Neumann et al. used an interferometric approach to measure the distance between an optically-trapped 500-nm bead and the interface at distances between 1 and 3 *μ*m [4]. Another method involves measuring the apparent thickness of a thick fluorescent film, but the smallest film thickness measured was 15.8 *μ*m [5]. A third method, similar to the *xz*-slice method presented here, measures the apparent axial size of fluorescent beads. These studies typically use beads that are 6–10 times the size of an *E. coli* cell, or are 50–100 *μ*m deep within a sample [6–9]. The majority of these methods used moderate NA objectives (1.2–1.3) and confocal laser scanning systems. Here, we present two experimental methods for measuring the apparent focal shift for very high numerical objective systems (≈ 1.49) in an epifluorescence modality operating very close to the *n*_1_/*n*_2_-interface (≈ 0–1.5 *μ*m). We also introduce a numerical approximation for situations where measurement is impractical.

## Materials and Methods

### Imaging system

The imaging system that we used is a custom built microscope. It includes a monolithic block of aluminum which holds the microscope objective (primarily a Nikon CFI Apo TIRF 100X Oil), long travel *x*, *y*, *z* stages for sample positioning (Newport 562-XYZ with HR-13 micrometers) and short travel piezo stage for fine positioning and *z*-stacks (Physik Instrumente P-611.3 NanoCube). Illumination is provided by a series of lasers (Vortran Stradus 405 and 488 nm, CrystaLaser 561nm) and images are recorded on an EMCCD (Andor DU-897). The system is controlled by custom software (National Instruments Labview 2012).

### MultiBead: Estimating focal shift using mixtures of different sized fluorescent microspheres

The first experimental method determines the relative focal shift of a system by imaging mixtures of fluorescent microspheres of different sizes and colors. Dilute suspensions of beads are mixed together, bound to a 22 mm square #1 thickness coverslip and covered with a standard microscope slide. The concentration of beads was adjusted to yield roughly 15 to 100 beads per 1600 *μ*m^2^ field of view. Most of the beads started as stocks of 1% solids and were diluted by a factor of 100 to 10,000. In practice, the beads are diluted twice during sample preparation. The manufacturer’s stock is first diluted by a factor of 10 or 100. The second dilution happens as the sample is prepared, 1–5 *μ*L are added to 25 *μ*L of water. To allow the beads to stick to the coverslip, 0.5 *μ*L of 200 mM MgCl_2_ (final concentration 3–4 mM) were added to this dilute bead solution and vortexed. Immediately following the addition of the salt, the sample is placed between a slide and coverslip. Allowing the sample to sit after adding salt leads to signficant aggregation of the beads. The choice of bead sizes reflects the normal operating range of our experiments; we chose beads with diameters between 50 nm and 2 *μ*m and a few different colors (Dragon Green and Flash Red from Bangs Labs, FC02F, FC03F, FC04F; 565/580 from Life Technologies, F8794; TetraSpeck from Life Technologies, T7279). For each of the combinations of beads of different sizes and colors, stacks of images were taken at spacing of 50 or 100 nm covering a range of 5 to 10 *μ*m. To prevent the samples from drying out, they were sealed with a 1:1:1 mixture petroleum jelly, lanolin, and paraffin.

**Fig 2:**
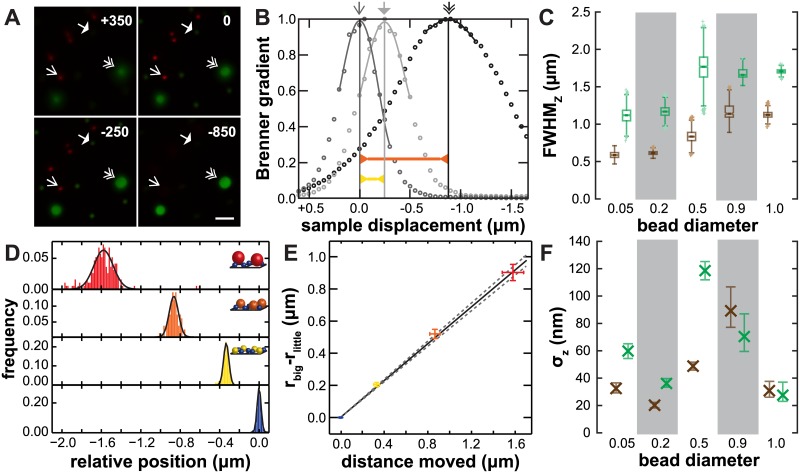
Relative focal shift as measured as the distance between beads of different sizes. (A) Example images with a sample of three types of beads (100 nm TetraSpeck, 510 nm Dragon Green, 1040 nm Dragon Green) are shown at different sample positions. (B) Brenner gradients for three example beads highlighted in panel A. (C) Box and whisker plots of the full-width at half-maximum Brenner gradient (brown) and peak intensity (green). (D) Histograms of relative distances between beads and the plane of the smallest beads. (E) Sample motion needed to refocus from one size bead to another is proportional to the difference in their sizes. *α* is the slope of this line. Colors as in panel D. *x* error bars are standard deviations of observed positions. *y* error bars are 5% relative deviation in bead diameter. Solid line is the fit *y* = *αx* and the dashed lines are the 95% C.I. for *α*. (F) Standard deviation of bead positions, measured by Brenner gradient (brown) and peak intensity (green). 99% C.I. for *σ* is calculated from a bootstrap analysis and displayed as the error bars.

We used a set of image processing algorithms to measure bead positions in the *z* direction. First, *xy* positions for the beads were determined as peaks in a standard deviation projection along the focal axis. Square regions around each of the peaks were identified and the Brenner gradient was calculated for each *z* position of the sample. This metric is commonly used for auto-focus routines in a variety of microscopy modalities [10, 11]. The *z* position at which the bead was in best focus was determined with sub-step precision by fitting the Brenner gradient profile to a degree-four polynomial in the 1 *μ*m region surrounding the peak value (see Fig 2B). Another focus metric is the peak pixel intensity. As seen in Fig 2C, the full-width at half-maximum (FWHM) of the Brenner gradient is narrower along the focal dimension than the peak pixel intensity. Using the peak pixel intensity metric instead of the Brenner gradient results in similar, but less precise, estimates of the focal shift.

This process of peak detection was repeated on each color channel. To correct for small tilts of the sample relative to the optical axis, a plane was fit to the (*x*, *y*, *z*) positions of the smaller set of beads. We analyzed the difference in bead heights for the other sized beads relative to this plane. This plane is approximately 25–100 nm from the true coverslip-water boundary. This tilt correction is extremely important. Because we are measuring positional accuracy to better than 50 nm across a 40 *μ*m by 40 *μ*m field of view, the sample would need to be oriented correctly with roughly 1 arcmin of precision. In practice, the observed tilt of the plane is on the order of 5 arcmin. This is likely due to a mechanical tilt between the mechanical screw in the microscope stage and the optical axes of the objective.

Following determination of the *z*(*x*, *y*) = 0 plane, the *z* positions of all the beads were calculated. These are pairwise distances, that is, the distance one must move the sample to move the focal plane from the center of one bead to the center of another. The distribution of these relative distances is plotted as probability density functions (PDFs) in Fig 2D with normal distribution fits to the data. Fig 2E is a plot of the means of these measurements. The distance the focal plane moved in the sample was calculated as simply the difference in the diameters of the beads. This is one way to represent the relative focal shift, to move the focal plane from one plane of beads to another, one must move the sample *α* ^−1^ times as far. In our system, *α*_*multiBead*_ = 0.57±0.02. We also show below that this multiBead method can be used over a wide range of *n*_2_ and NA.

### XZSlice: Estimating focal shift using *xz* slices of beads and cells

**Fig 3:**
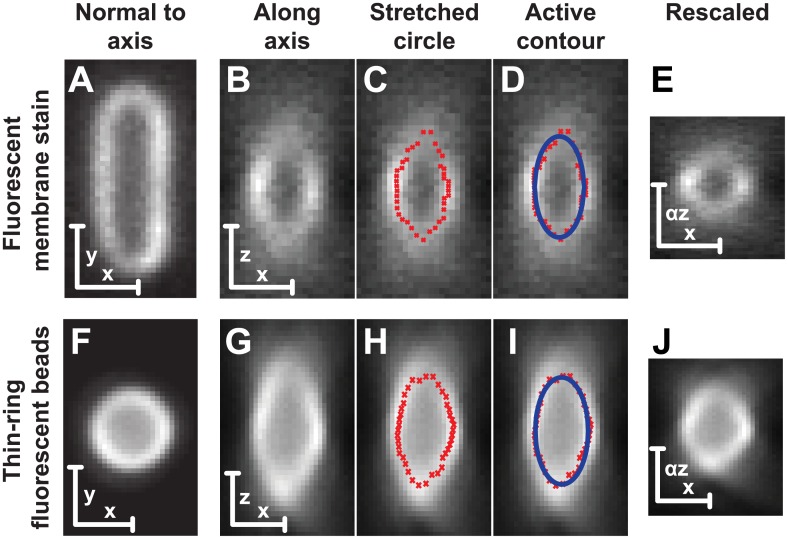
Fluorescent images showing the disparity between slices in a single focal plane and along the focal dimension. Scale bars are 1 μm. (A-E) *E. coli* cell stained with a membrane dye (FM 4–64). (F-J) 1 *μ*m sphere with a fluorescent ring stain. (A,F) *xy* slice (B,G) *xz* slice showing the apparent elongation of the object along the focal axis. (C,H) Active contour fit to the ridge of maximal intensity in red. (D,I) Stretched circle fit in blue *r* ^2^ = (*αz*)^2^ + *x* ^2^. (E,J) *xz* slice shown after scaling *z* by *α*.

A second method to measure the relative focal shift of a system uses fluorescent, ring-stain microspheres. These microspheres have fluorescent dye only in a thin shell at the surface of the sphere (Molecular Probes 1.0 *μ*m FocalCheck beads, F14791). From a 3D image of one of these beads, *xy* and *xz* slices are generated. As seen in Fig 3, these slices show a fairly clear ridge of peak intensity. Even though the beads are quite spherical, these slices appear extremely extended along the *z* dimension due to the focal shift of the system.

To extract the focal shift, the ridge of intensity is fit using an active contour method [12]. A stretched circle yields a good fit to the points of this active contour *r* ^2^ = (*x* − *x*_0_)^2^ + (*α*(*z* − *z*_0_))^2^. This process does not require any intervention from the user and the calibration can therefore be done rapidly on hundreds of beads. For our system using 1 *μ*m spheres, *α*_*ringSphere*_ = 0.59±0.02. This method of fitting cross sections through fluorescent rings can also be applied to cylindrical or spherical cells with fluorescent membranes. Fig 3 shows cross-sections through *E. coli* stained with the membrane dye FM 4–64. Using the same stretched circle fit to an *xz* slice, *α*_*cellMembrane*_ = 0.52±0.04. We show in Fig 3E and 3J the resulting image when this calibration is applied as a postprocessing step. Correcting the spacing of the pixels shows a nearly circular cross section in the sample. The difference in the size and shape of the microscope’s point spread function along *xy* and *z* is still visible. Along *xy*, the bead appears symmetric and circular. Along *xz* the larger size of the PSF and the first Airy rings can be clearly seen.

### Polynomial extrapolation: Estimating focal shift during design

**Fig 4:**
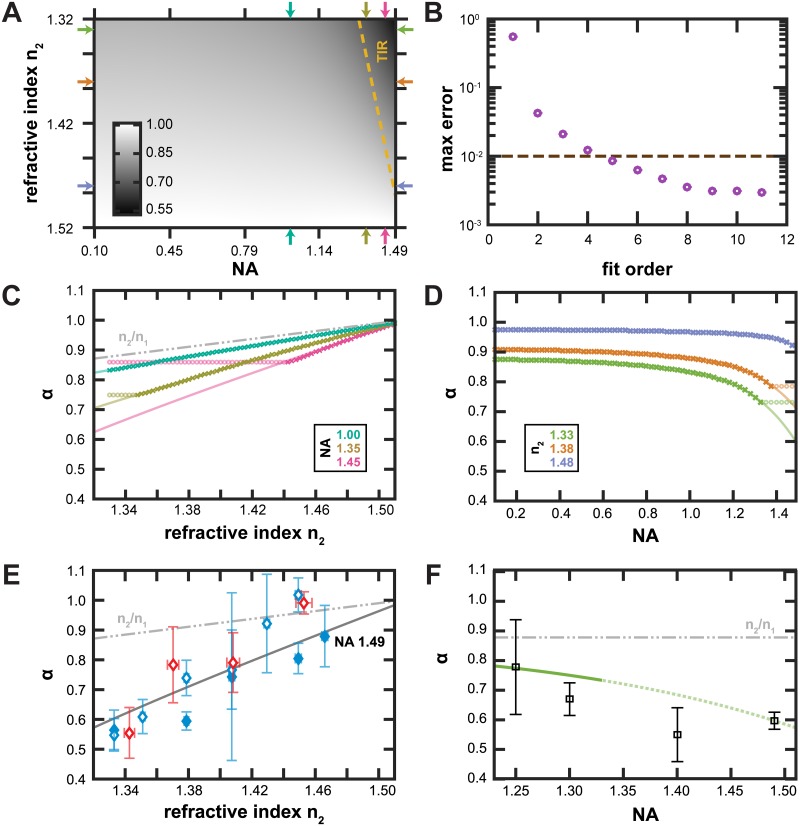
Polynomial extrapolation of relative focal shift as a function of NA and *n*_2_. (A) *α*_*poly*_ as a function of NA and *n*_2_. The colormap (inset) gives the value of *α*. The TIR region is marked by a dashed gold line. Colored arrowheads indicate where the 1D slices through this surface are taken for panels C and D. (B) The order of the polynomial extrapolation is chosen as the lowest order whose maximal error (grape) is less than the maximal difference between *α*_646_ and *α*_489_ (cocoa). (C-D) *α* evaluated by the theory in [2] at various values of NA and *n*_2_ (symbols) along with the global fit to Eq 3 (lines). Symbols that corresponding to TIR systems are shown as faded circles. (C) *α* as a function of refractive index for three different values of numerical aperture (1.00 in turquoise, 1.38 in olive, 1.48 in orchid). Also included is the *n*_2_/*n*_1_ approximation (gray—⋅⋅). (D) *α* as a function of numerical aperture for three different values of *n*_2_ (1.33 in pistachio, 1.38 in goldenrod, 1.43 in periwinkle). (E) Experimentally observed *α* for imaging system of varying refractive index. Refractive index of the medium was increased by including glycerol (cornflower) or sucrose (dark red). Two copies of the same model of objective were measured, results from one are filled symbols and the other are open. Evaluating the *α*_*poly*_ with an NA of 1.49 is shown in charcoal. *α* error bars are 90% CI. *n*_2_ error bars reflect changes in additive concentration by ± 1.5%. (F) Experimentally observed *α*_*multiBead*_ as a function of NA for imaging systems with various objectives using the multiple bead sizes method.

While there have been multiple attempts to model the focal shift associated with objectives of moderate (1 < NA < 1.3) and high numerical aperture (1.3 < NA), rays greater than the critical angle are ignored in the transmitted direction. This model can still be of great use for estimating the relative focal shift of a system when measurement is impractical, such as during design. The simplest approximation is a relative focal shift of *n*_1_/*n*_2_, the paraxial ray approximation [2, 7]. As shown in Fig 4C, this approximation is useful for systems with low numerical aperture.

Perturbations from this limit occur for high numerical aperture systems and systems that image close to the plane interface. For systems below the critical angle, *α* can be estimated using the method of Wiersma [2]. We have extended this model above the critical value by fitting the whole 2D plane of *α*(NA, *n*_2_) to an order-six polynomial in two dimensions.

$$\begin{array}{c} \alpha (\text{NA},n_{2})=\sum_{j,k=0}^{j+k\leq 5}A_{jk}\cdot {\text{NA}}^{j}\cdot {n_{2}}^{k} \end{array}$$ (3)

Additionally, we force *A*_00_ = 1 and *A*_01_ = −1/*n*_1_, corresponding to the *n*_2_/*n*_1_ limit. This polynomial is then fit globally for all NA and *n*_2_ below the TIR limit. As seen in Fig 4C-4D, this polynomial has sufficient terms to accurately reproduce the entire surface. The root mean squared deviation between the surface and the data was about 0.001. Although a theoretical functional form of *α*(*NA*, *n*_2_) has not been worked out, we choose to use a polynomial fit as the form for the extrapolation. Extrapolation can be fraught with errors, especially when the largest slopes and curvatures are near the edge of the range used to fit, as is the case here. In order to estimate which order polynomial we should use, we compared the theoretical prediction of *α* for two different wavelengths which span the color range that we used, *λ*_*low*_ = 486 nm and *λ*_*high*_ = 646. For these calculations, we used the wavelength dependent refractive index for our immersion oil (Cargille LDF). These two surfaces (*α*_486_ and *α*_646_) are similar with an root mean squared difference of 0.0065 and a maximal absolute difference of 0.0110. We set this maximum distance as the cutoff for our polynomial fit. That is, we chose the lowest order polynomial such that maximum distance from the fit surface to the theoretical surface is less than 0.01. This maximal distance is also a factor of two smaller than the error estimate for our experimental precision, setting this as a conservative lower bound for the order polynomial order to use, in our case, fifth order.

This polynomial expansion can be evaluated for imaging systems that are capable of TIR, something that the theory of [2] is unable to do. To test the validity of this extrapolation, we measured the focal shift in a variety of refractive indices. To increase the refractive index of the medium, we made solutions from 15–95% w/w glycerol and 7–65% w/w sucrose and used these as the mounting media. These correspond to refractive indices from 1.343 to 1.466 [13, 14]. These solutions were used for both dilutions of the beads: from the manufacturer to a working stock and in the final sample. We included additional sonication steps after each dilution to ensure that beads in these viscous samples were well isolated. The manufacturers ship the beads in water, leading to a small additional contribution of water in the final sample. The largest effect was samples with 1 *μ*m beads. These had an excess 1.5% w/w of water. This small contribution to the refractive index, between 0.001 and 0.007, is shown as the horizontal error bars in Fig 4E.

## Results

### Precision in determining *z* position

We estimate our precision in determining the positions of the center of beads by the width of the distribution of bead positions (Fig 2F). This width, 20–40 nm for the smallest beads, increases as the beads increase in size. As beads get larger, the absolute error associated with the difference in bead size and position determination are both likely to increase. Assuming an entirely monodisperse population of beads as a worse case estimate, our position determination is typically better than 50 nm. Instead of using the standard error of the mean, we use this width as a more conservative estimate for our error estimates of observed focal shift. This estimate of precision is somewhat larger than the manufacturer estimated variation in bead diameters (1%–5%).

### Linearity in *α*

**Fig 5:**
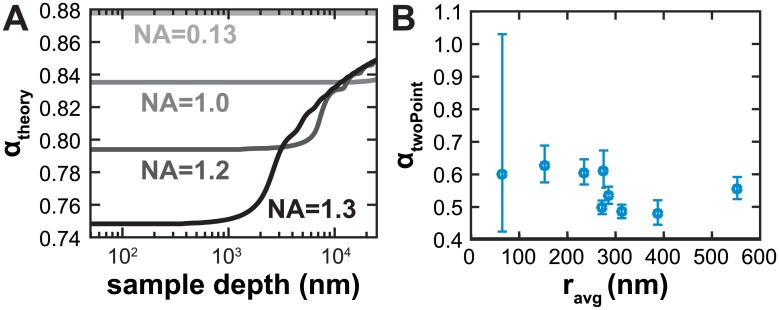
Constant *α* near the the coverslip. (A) Apparent *α* for various sample depths as predicted by Eqs 1–2. (B) *α*_*twoPoint*_ plotted against the average radius of the two bead sizes that went into the measurement.

The method of [2] predicts that *α* should be nearly independent of sample depth, but with an increase toward the *n*_2_/*n*_1_ limit as the sample depth gets large (Fig 5A). This is consistent with the notion that as the ratio of the sample distance to focal length of the objective increases, the effective NA of the system decreases. For a nominal 1.3 NA system, theory predicts that this increase in *α* sets in around a few μm, a similar the depth that was used in [4]. Because the multiBead method samples the focal shift over a range of sample depths we calculated an apparent *α*_*twoPoint*_ from each bead pair sample and plot it as a function of the average radius of the two beads (Fig 5B). We use this as a proxy for the depth into the aqueous sample as the beads are adhered to glass surface. The observed values of *α*_*twoPoint*_ are fairly constant over our experimental range.

These measurements are noisier than estimating the slope of all the bead combinations aggregated together, particularly at very small differences in bead sizes. The distributions of bead positions are close to normally distributed so we use the mean position ± one standard devidation as our error estimate on *α*_*twoPoint*_. Similar positional precision across the size ranges leads to large fractional error at small differences in bead sizes. For example, 200 nm diameter beads appear 136 nm ± 57 nm away from a plane of 50 nm diameter beads giving an *α*_*twoPoint*_ of 0.6 with an error range from 0.4 to 1.0.

### Testing the validity of extrapolation

In Fig 4E, we explore the impact of refractive index on relative focal shift. In the range of experimentally obtainable media between pure water and pure glycerol, the polynomial extrapolation appears to be a rather good proxy for the focal shift. However, at some point, the polynomial extrapolation must fail. In fact, it predicts that the focal shift will cross 0 and go negative for embedding media with *n*_2_ close to 1, clearly an unphysical result. Whenever possible, it is best to measure the focal shift of the imaging system being used. As seen by the difference in the open and closed symbols in Fig 4E, two different copies of the same objective can behave somewhat differently. This is likely due to the particular aberrations present in each.

In Fig 4F, we apply the multiBead method to objectives of a lower numerical aperture (Table 1). As the numerical aperture goes down, the focal shift goes up. The pistachio colored line for the polynomial extrapolation switches from solid to dotted when reaching the critical angle. Because the multiBead methods measures the distance between two different colors of beads, it is important that the imaging system be free from longitudinal chromatic aberrations. An imaging system that focuses different colors at different positions will lead to errors in estimating the true distance between two sizes of beads. One way to estimate the severity of chromatic aberration is to measure the observed distance between focusing on multi-colored beads in various color channels. For our measurements of different objectives, we tested to ensure that the variation in focal plane between different filter sets was less than 30 nm. For one objective, the Nikon Plan 1.25 NA objective, the chromatic aberrations were very severe (> 200 nm). For this objective, we used a modified version of the multiBead approach using sets of beads of the same color. To ensure that we could tell the types of beads apart, they needed to be of a greater difference in size. This modification lead to a reduced number of samples and a reduced precision in measuring *α*.

**Table 1:** Properties of objectives used to test the versatility of the multiple bead method.

**Manufacturer**	**Type**	**Numerical Aperture**	**Magnification**
Nikon	CFI Apo TIRF 100X Oil	1.49	100X
Nikon	CFI Plan Apo VC 100X Oil	1.40	100X
Zeiss	Plan Neufluar 100X	1.30	120X
Nikon	CFI Plan DL 100X Oil	1.25	100X

### Comparison of methods

All three methods presented here produce very similar estimates of about 0.57 for the apparent focal shift of a 1.49 NA objective imaging into water (Table 2). These three methods are not equally applicable in all situations. The polynomial extrapolation requires no physical imaging system and is therefore practical even during instrument design. Fitting *xz* slices has difficulties in the assumption that the object really has cylindrical symmetry and the image processing requirements are high.

**Table 2:** Summary of a variety of methods used to measure relative focal shift. Values are reported for our highest NA objectives, NA 1.49, imaging into an aqueous environment, *n*_2_ = 1.33.

**Method**	**Apparent focal shift**	**Ease**	**Accuracy**	**Caveats**
*n*_2_/*n*_1_	0.88	+++	- -	
Theory from [2]	0.73	++	++/-	Accuracy limited for TIR objectives
Polynomial extrapolation	0.59	+	+	Ignores objective specific aberrations
Multiple bead sizes	0.60 ± 0.03	+	++	
Cross sections: ring-stain beads	0.59 ± 0.02	-	++	
Cross sections: single rod-like cells	0.52 ± 0.04	- -	++	

## Discussion

The ability to accurately and easily calibrate the focal shift of a microscope’s imaging system is an important aspect to properly measuring 3D intensity distributions. The two experimental methods described here report similar values of focal shift for our particular imaging system (see Table 2). Measuring the focal plane for different sized fluorescent beads is the simplest experimental technique. Like the other methods, this gives an absolute measure of focal shift. Using multiple combinations of bead sizes, easily obtainable from commercial sources, allows the investigator to measure focal shift over her entire observation range. The beads can also be embedded in a medium whose refractive index closely matches the experimental situation. This is a great improvement over the other methods, where looking at a variety of size scales is more difficult. The ability to measure across the entire range of sample sizes is also important to assess the linear assumption of the (Δ*z* − *F*) = *α*Δ*z*. Slices through ring-stained beads also afford a moderate range of sizes, although not as diverse as simple fluorescent beads. Unfortunately the ring-stain beads and membrane stained rod-shaped cell methods are more difficult to implement because they require more complicated image processing techniques. In addition, the point spread function of many objectives, especially TIR objectives, are highly asymmetric along the focal dimension. This leads to significant and asymmetric blurring of images along the optical axis. In other words, although it is usually obvious which *z* plane is the center plane of bead, the cross-section of the bead may not even appear to be a closed contour. Sometimes the difference in intensity at the top and bottom of an *xz* cross-section gives the bead an appearance of an asymmetric diamond ring.

For some applications, the impact of focal shift can be diminished by using a lower numerical aperture objective or index matching the sample to that of the imaging system [15]. Stochastic super-resolution microscopy techniques often utilize high NA objectives to collect a large fraction of emitted photons and work close to the coverslip sample interface [16–18]. *xyz* coordinates of the single fluorophores can be extracted using a variety of 3D single molecule techniques, yielding a full three-dimensional intensity distribution of the sample [15, 19, 20]. Properly correcting for focal shift in these samples becomes an essential component in experimental design [15], or in data analysis and reconstruction [21–23]. Even for wide-field fluorescence, improper accounting of focal shift can lead to improper measurements of the size and shape of objects [7]. Correcting for focal shift is also essential when working with mixed imaging modalities, for example confocal imaging and x-ray diffraction of single crystals for structural biology [24].

## Conclusion

Here we present three simple methods for measuring the relative focal shift of a microscope system very near the glass sample interface. The first is to measure the sample displacement required to move the focal plane between beads of different known sizes. The second to measure an object with shell-like fluorescence, such as a ring-stained bead or membrane-stained bacterial cell. From this sample, one can measure how stretched or squashed the circular xz cross-section appears. The final method utilizing a polynomial extrapolation to estimate focal shift in a system where measurements are not possible. In addition to introducing these methods, we cross-validated them and showed that they could be used in optical configurations including a variety of objectives and refractive indices. For our NA-1.49 system, the apparent focal shift when imaging into aqueous solution is about 0.6, significantly lower than the typical *n*_2_/*n*_1_ approximation.

## Supporting Information

S1 Dataset Electric field as a function of *z* position as calculated from Wiersma et al. [2]. In each sub-table, the first column is the coordinate along the *z* axis. The next three columns are the real, imaginary and magnitude of the electric field. Six sub-tables for positions of the *n*_2_/*n*_1_ interface (d) relative to the focal length of the objective (f). Three header rows, 1000 data rows.
(CSV)

S2 Dataset Displacements of peak position due to focal shift as calculated from Wiersma et al. [2]. In each sub-table the first column is the amount of sample motion Δ*Z*. The second column is the additional motion of the peak position of the electric field. See Fig 1 for a schematic. Four sub-tables for NA = 0.13, 1.0. 1.2 and 1.3. Two header rows, 135 data rows.
(CSV)

S3 Dataset FWHM and bead center variation for a variety of sizes and colors of beads. First column, full-width at half maximum for peak pixel intensity as a function of *z*. Second column, full-width at half maximum for Brenner gradient as a function of *z*. Third column, relative position of the bead based on peak Brenner gradient. Fourth column, relative position of the bead based on peak pixel intensity. Fifth column, the diameter of the bead. One header row, 3069 data rows.
(CSV)

S4 Dataset Observed relative positions of beads. Four different sizes were measured relative to the plane made by the smallest bead in the sample. One header row, 65–398 data rows.
(CSV)

S5 Dataset Average properties of the apparent distance between beads of different sizes. The first column is the size of the larger beads in the sample. The second column is the size of the smaller beads in the sample. The third column is the real space distance between the two radii. The fourth column is an estimate of expected deviation in bead sizes from 1% variation in bead diameters. The fifth column is the observed mean distance moved from focusing on one size bead to the other. The sixth column is the standard deviation of the observed distances. One header row, four data rows.
(CSV)

S6 Dataset Standard deviation in observed bead positions. This table includes measurements using Brenner gradient and peak pixel as the focus metrics. Bead sizes and focus metrics are marked as headers. One header column, one header row. Four data rows, ten data columns.
(CSV)

S7 Dataset Coordinates of outline from *xz* cross-section of bacterial cell stained with the membrane stain FM 4–64. Two sub-tables. First is an active contour fit to the peak intensity ridge in the image. The second sub-table is the best fit stretched circle to this active contour. In each sub-table, the first column is the row coordinate and the second is the column coordinate. Row and column are used instead of *xy* to distinguish the camera and imaging coordinate system from the real space coordinate system. Pixel sizes are 80 nm along both the rows and the columns of the image. One header row, 40 data rows.
(CSV)

S8 Dataset Coordinates of outline from *xz* cross section of a 1 *μ*m ring-stained polystyrene bead. Two sub-tables. First is an active contour fit to the peak intensity ridge in the image. The second sub-table is the best fit stretched circle to this active contour. In each sub-table, the first column is the row coordinate and the second is the column coordinate. Row and column are used instead of *xy* to distinguish the camera and imaging coordinate system from the real space coordinate system. Pixel sizes are 80 nm along both the rows and the columns of the image. One header row, 40 data rows.
(CSV)

S9 Dataset Focal shift as calculated from Wiersma et al. [2]. The first column is the refractive index (*n*_2_). The second column is the numerical aperture. The third column is the apparent relative focal shift *α*(*n*_2_, NA). One header row, 3750 data rows.
(CSV)

S10 Dataset Apparent focal shift measured using the multiBead method for samples embedded in various refractive indices. The first column is the focal shift, the second is half-size of the 90% CI. The third is a label for the objective (1: 1.49 NA objective A + glycerol, 2: 1.49 NA objective B + glycerol, 3: 1.49 NA objective B + sucrose). The fourth column is the refractive index of the solution. The fifth is the numerical aperture of the objective. The sixth is an estimate for the error in the refractive index based on a difference in the concentration of the additive by ± 1.5% w/v. One header row, 15 data rows.
(CSV)

S11 Dataset Apparent focal shift measured using the multiBead method for samples measured with different objectives. The first column is the focal shift, the second is half-size of the 90% CI. The third column is the refractive index of the solution. The fourth column is the numerical aperture of the objective. One header row, four data rows.
(CSV)

S12 Dataset Linearity of *α*_*twoBead*_. First column is the observed offset between two sizes of beads. The standard deviation of these distances is the second column. The third column is the number of beads in the sample. The fourth column is the diameter of the smaller beads. The fifth column is the diameter of the larger beads. The sixth column is the average radius of the two beads. This is used as the coordinate along the abscissa of Fig 5B. Columns 7–9 have the mean focal shift and the apparent focal shift calculated from the mean distance ± one standard deviation. One header row, nine data rows.
(CSV)
